# Clinical effects of Yiqi-Yangyin-Huoxue granules in the management of type 2 diabetes mellitus and early vascular aging: a randomized, double-blind, placebo-controlled trial protocol

**DOI:** 10.3389/fmed.2026.1768610

**Published:** 2026-06-03

**Authors:** Jinyi Fu, Yifei Qi, Jingyi Zhan, Linjing Su, Yucong Gao, Qingbing Zhou, Ying Zhang

**Affiliations:** 1 Institute of Geriatrics, Xiyuan Hospital, China Academy of Chinese Medical Sciences, Beijing, China; 2 Beijing Tsinghua Changgung Hospital, Tsinghua University, Beijing, China; 3 Qi-Huang College, Beijing University of Chinese Medicine, Beijing, China; 4 Key Laboratory for Preventing Vascular Aging by Combination of Disease and Syndrome, National Administration of Traditional Chinese Medicine, Beijing, China

**Keywords:** baPWV, clinical protocol, early vascular aging, traditional medicine, type 2 diabetes, Yiqi-Yangyin-Huoxue granule

## Abstract

**Background:**

Type 2 diabetes mellitus (T2DM) has become a major global public health challenge, affecting more than 500 million adults worldwide. Early vascular aging (EVA) is one of the key pathological changes for diabetic vascular complications. However, targeted and effective therapeutic strategies remain limited. Based on our previous research and clinical experience, we developed a granulated natural herbal formulation, Yiqi-Yangyin-Huoxue (YQYYHX). This trial aims to evaluate the clinical efficacy and safety of YQYYHX in patients with T2DM and EVA, and to preliminarily explore its potential mechanisms.

**Methods:**

This is a single-center, randomized, double-blind, placebo-controlled, parallel-group clinical trial. It will enroll 120 participants with T2DM and EVA, who will be randomized in a 1:1 allocation ratio to two groups. In addition to standard therapy, the treatment group will receive the YQYYHX granules, while the control group will receive a matched placebo. Based on mass spectrometry analysis, daidzin, salvianolic acid L, and oleamide were identified as the compounds with the largest peak area in YQYYHX. After a 12-week intervention, brachial-ankle pulse wave velocity (baPWV) is planned to be assessed as the primary outcome. Secondary outcomes are expected to include the ankle-brachial index, blood glucose levels, lipid profiles, inflammatory cytokines, 6-min walk test and questionnaires. Safety will be evaluated using liver transaminases, serum creatinine, and related indicators. Serum and fecal samples will be collected before and after treatment. The serum will be used for metabolomic and proteomic sequencing analysis, and feces will be used for metagenomic sequencing. Electronic case report forms are generated within the clinical record system, ensuring that all follow-up information is traceable. Subsequently, the data will be entered into a specific electronic data capture, and the data administrator will verify it.

**Results:**

The recruitment began on March 27, 2025 and is expected to end on December 31, 2026. As of March 10, 2026, 72 participants have been enrolled.

**Conclusion:**

This rigorously designed trial is expected to generate reliable evidence. As a complementary and alternative therapeutic option, YQYYHX has the potential to benefit patients with T2DM and EVA.

**Clinical trial registration:**

This trial has been registered with the International Traditional Medicine Clinical Trial Registry Platform (http://itmctr.ccebtcm.org.cn/, ITMCTR2024000388).

## Introduction

In 2024, about 588.7 million people worldwide were living with diabetes (1); by 2050, this number will increase to 1.31 billion (95% UI, 1.22–1.39 billion) (2). The prevalence of type 2 diabetes mellitus (T2DM) and its complications has shown an overall increasing trend (3), while the average age at diagnosis appears to be decreasing (4, 5), contributing to heavy burdens on the society and affected individuals. Diabetic vascular complications include macrovascular and microvascular diseases, which are closely interrelated (6).

As a physiological process, vascular aging represents the age-associated degenerative modifications in vascular structure and function. In contrast, early vascular aging (EVA) is a pathological state and represents a key pathophysiological foundation for vascular complications in T2DM (7). The primary pathological manifestations of EVA are atherosclerosis and arterial stiffness. Atherosclerosis of the lower extremity arteries may progress to lower extremity arterial disease (LEAD), diabetic foot or other diseases. The prevalence of LEAD is 21.2% in T2DM patients (8). Diabetic individuals have twice the risk of developing LEAD compared with non-diabetic individuals (9), and the disease progression is faster and more severe. For LEAD, cilostazol may improve intermittent claudication through anti-platelet, anti-inflammatory and vasodilatory effects (10), and severe cases require interventional therapy or even amputation (11).

Our goal is to prevent EVA through pharmacological interventions before the development of LEAD. However, targeted and effective drugs to prevent EVA remain quite limited (12). Traditional Chinese medicine (TCM) offers unique advantages through its multi-target and multi-pathway mechanisms. Clinically, it can effectively alleviate symptoms and deserves further investigation. Based on our previous research and traditional theories, we developed Yiqi-Yangyin-Huoxue (YQYYHX) formulation and formulated it into granules. It contains *Panacis quinquefolii* radix, *Chuanxiong* rhizome, *Paeoniae radix rubra*, and other herbs, traditionally used to reinforce qi, nourish yin and promote blood circulation. It has been used in real-world practice and observed to effectively alleviate symptoms such as coldness, numbness, distension, and pain in the diabetic lower extremities.

We hypothesize that YQYYHX may have the potential to prevent or slow the progression of EVA in patients with T2DM. To examine this possibility, we have designed a clinical trial to generate high-quality evidence, assess the clinical efficacy and safety of YQYYHX granules in patients with T2DM and EVA, and explore potential underlying mechanisms (13, 14).

## Methods

### Study design

**Figure 1:**
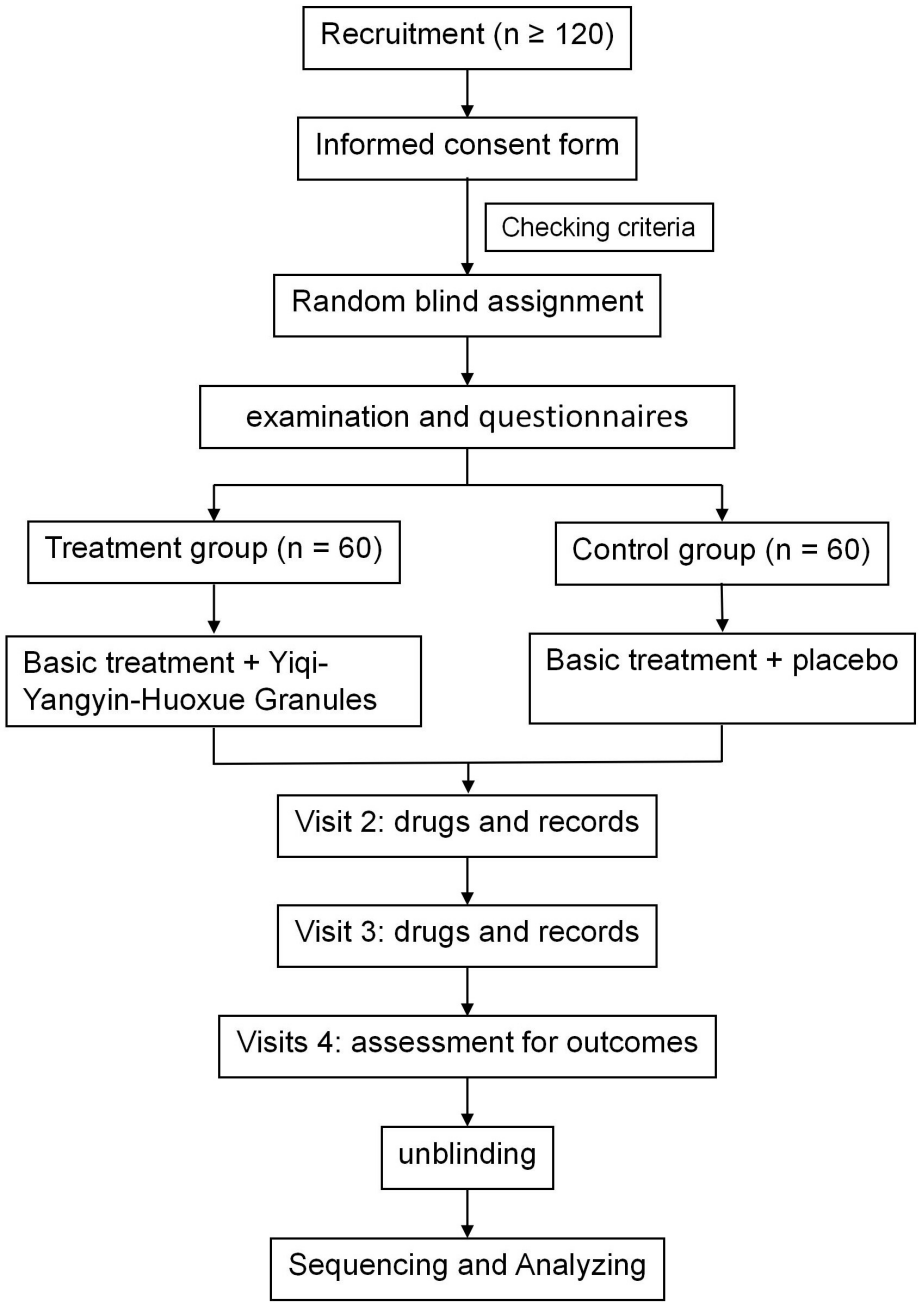
Study flowchart.

This study is a single-center, randomized, double-blind, placebo-controlled, parallel-group clinical trial. The trial is designed in accordance with contemporary SPIRIT guidance (15). The SPIRIT schedule is shown in Table 1, and the study flowchart is shown in Figure 1.

**Table 1:**
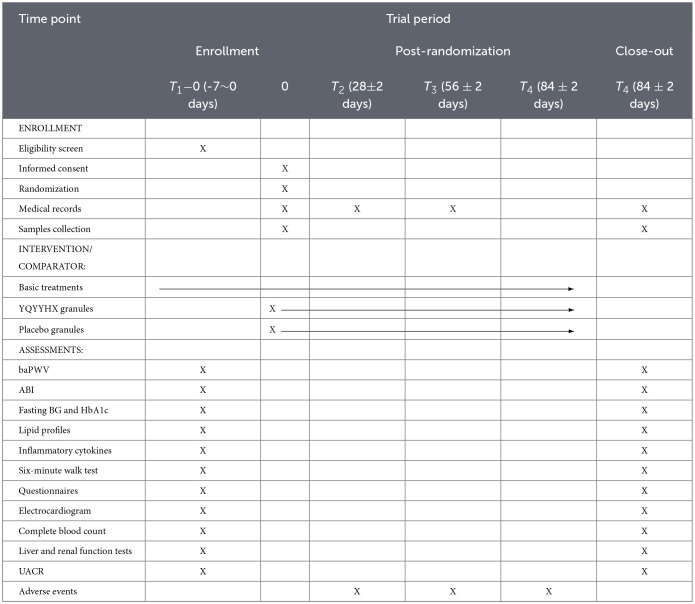
Study period.

The lipid profiles include TC, total cholesterol (TC); TG, triglycerides; HDL-C, high-density lipoprotein cholesterol; LDL-C, low-density lipoprotein cholesterol. The questionnaires used in this study include vascular quality of life questionnaire-6 (VascuQol-6), a symptom questionnaire, and a questionnaire of traditional Chinese medicine syndrome. The liver function tests include TP, total protein; Alb, albumin; ALT, alanine aminotransferase; AST, aspartate aminotransferase; GGT, γ-glutamyl transferase; TBA, total bile acids; T-Bil, total bilirubin; D-Bil, direct bilirubin. The renal function tests include urea; Cr, creatinine; UA, uric acid; HbA1c, Glycated hemoglobin; YQYYHX, Yiqi-Yangyin-Huoxue formulation; baPWV, brachial-ankle pulse wave velocity; ABI, ankle-brachial index; BG, blood glucose; UACR, urinary albumin-to-creatinine ratio.

The trial has been approved by the Ethics Committee of Xiyuan Hospital, China Academy of Chinese Medical Sciences, Beijing, China (No. 2024XLA091; version 3.2, October 14, 2025). The committee will review major protocol amendments and conduct annual monitoring of the study. The trial has been registered on the International Traditional Medicine Clinical Trial Registry Platform (http://itmctr.ccebtcm.org.cn/, ITMCTR2024000388), and is in the process of being registered on the National Health Security Information Platform for Medical Research Registration and Record System (https://www.medicalresearch.org.cn/).

### Participants

Recruitment will be conducted at Xiyuan Hospital in Beijing, China. It began on March 27, 2025 and is expected to end on December 31, 2026. Participants will be identified through routine clinical care, public advertisements such as posters, and screening from the clinical database. All participants must strictly meet the inclusion and exclusion criteria, and voluntarily sign the written informed consent, including consent for specimen retention.

Experienced clinicians will assess eligibility based on their physical status. Research assistants will regularly communicate with them to enhance their adherence to the study.

### Inclusion criteria

1. Aged 40–85 years, regardless of sex.
2. Meets the diagnostic criteria for T2DM.
3. Evidence of lower extremity atherosclerotic plaques or stenosis, confirmed by Doppler ultrasound, CT angiography, or conventional angiography.
4. Fontaine stage I–IIA.
5. Meets the diagnostic criteria for EVA.
6. Differentiated as “Qi-Yin deficiency with blood stasis” syndrome in TCM.
7. Willing to sign the informed consent form.

### Exclusion criteria

1. Presence of other conditions affecting lower extremity function, such as autoimmune-related vasculopathies, arterial embolism, lumbar spinal stenosis, or similar disorders.
2. Presence of malignancy, thyroid or parathyroid disorders, hematologic diseases, acute decompensated chronic heart failure, or acute left-sided heart failure.
3. Altered mental status, cognitive impairment, or inability to communicate effectively.
4. History of lower extremity revascularization surgery or arterial stent implantation within the past year.
5. Significant hepatic dysfunction (aminotransferase ≥ 3 times the upper limit of normal) or renal dysfunction (serum creatinine ≥ 1.5 times the upper limit of normal) within the past 3 months.
6. Presence of absolute contraindications to the 6-minute walk (6MW) test.
7. Known allergy or contraindication to any drugs or components in this study.
8. Participation in another clinical trial in the past 3 months, or concurrent participation in another clinical trial.

### Diagnostic criteria

1. Diagnostic criteria for T2DM

In the presence of classic symptoms of hyperglycemia, such as polyuria, polydipsia, polyphagia, or unexplained weight loss, the diagnosis of T2DM can be established when any one of the following criteria is met (16):

Random blood glucose (BG) ≥ 11.1 mmol/L;

Fasting BG ≥ 7.0 mmol/L;

2-hour BG ≥ 11.1 mmol/L during a standard oral glucose tolerance test;

Glycated hemoglobin (HbA1c) ≥ 6.5%.

2. Diagnostic criteria for EVA

Age-adjusted pulse wave velocity (PWV) exceeds the upper limit of normal for healthy individuals by more than two standard deviations (17, 18).

3. Fontaine stage I - IIA classification

Stage I: Asymptomatic.

Stage IIA: Mild intermittent claudication; walking distance > 200 meters (19).

4. Diagnostic criteria for Qi-Yin deficiency with blood stasis syndrome

“Qi-Yin deficiency with blood stasis” syndrome can be diagnosed when either two primary symptoms or three secondary symptoms are present, accompanied by characteristic tongues and pulses.

Primary symptoms: Dry throat and mouth; shortness of breath with reluctance to speak; limb numbness; stabbing pain in the extremities.

Secondary symptoms: limb weakness; spontaneous or nocturnal sweating; heat sensation in the palms and soles; palpitations and insomnia; rough or dry skin; purplish lips; constipation or diarrhea.

Tongue and pulse characteristics: Pale-dark tongue or presence of ecchymoses; tortuous cyanotic sublingual veins; white coating. Pulse may be thin, deep, wiry, or choppy.

5. Absolute Contraindications for the 6MW

Unstable angina and myocardial infarction during the previous month (20).

### Randomization, allocation and blinding

The clinical research methodology team generated a simple randomization list using the block-randomized method with SAS 9.4, applying the allocation ratio of 1:1. Blinding of the investigational drugs was performed by statisticians and independent third-party personnel not involved in the trial.

Participants are assigned random numbers and corresponding drug codes sequentially according to their enrollment order. The blinding codes are stored confidentially in sealed envelopes until the end of the trial. Both the investigators and participants are blind to treatment allocation. If a serious adverse event happens, the principal investigator will request an emergency unblinding envelope from the Good Clinical Practice (GCP) office.

### Sample size

The mean ± SD of baPWV among T2DM patients was 1548.87 ± 305.48 cm/s in Beijing, China (aged 20–80 years) (21), 1580 ± 324 cm/s in Nanchang, China (aged 17–75 years) (22), and 1534.2 ± 254 cm/s in Kagoshima, Japan (aged 22–84 years) (23). Among healthy people, it was 1200 ± 150 cm/s (aged 40–49 years), 1390 ± 190 cm/s (aged 50–59 years), 1630 ± 300 cm/s (aged > 60 years) in Xinjiang, China (24).

Considering that the above data were derived from small-sample studies and the age ranges were not consistent with this study, we hypothesized that the baPWV was 1600 ± 260 cm/s for T2DM patients aged 40–85 years, and YQYYHX was expected to reduce it to 1440 ± 234 cm/s (25, 26). A change of 100 cm/s may represent a minimal clinically important difference (27). This study is designed by superiority, and the ratio between the treatment and control groups is 1:1. With a two-sided α = 0.05 and β = 0.1, the sample size is calculated to be 52 in each group using a *t*-test allowing unequal variances in PASS 15. The formula is as follows.


$$\begin{array}{l} n=\frac{{\left(Z_{1-\alpha /2}\;+\;Z_{1-\beta }\right)}^{2}\cdot 2\sigma^{2}}{{\left(\mu_{1}-\mu_{2}\right)}^{2}} \end{array}$$

Considering a 10%−15% dropout rate, 60 patients are needed in each group and the total sample size is 120.

### Intervention

Basic treatments include metformin, acarbose, insulin, statins, evolocumab, and other standard medical therapies. It is not fixed, individualized plans will be developed by the attending clinicians before the trial, to maintain HbA1c below 8.0% (28) and LDL-C below 1.4 mmol/L (29). Basic treatments will remain unchanged and other TCM therapies are prohibited during the trial.

Participants will take YQYYHX granules or placebo granules for 12 weeks. They should take it 30 min before meals or other medication, at a dose of 12 g per administration, twice daily. The placebo granules contain 10% inert ingredient and 90% excipient (dextrin), with a similar shape, color, and odor as YQYYHX granules.

**Figure 2:**
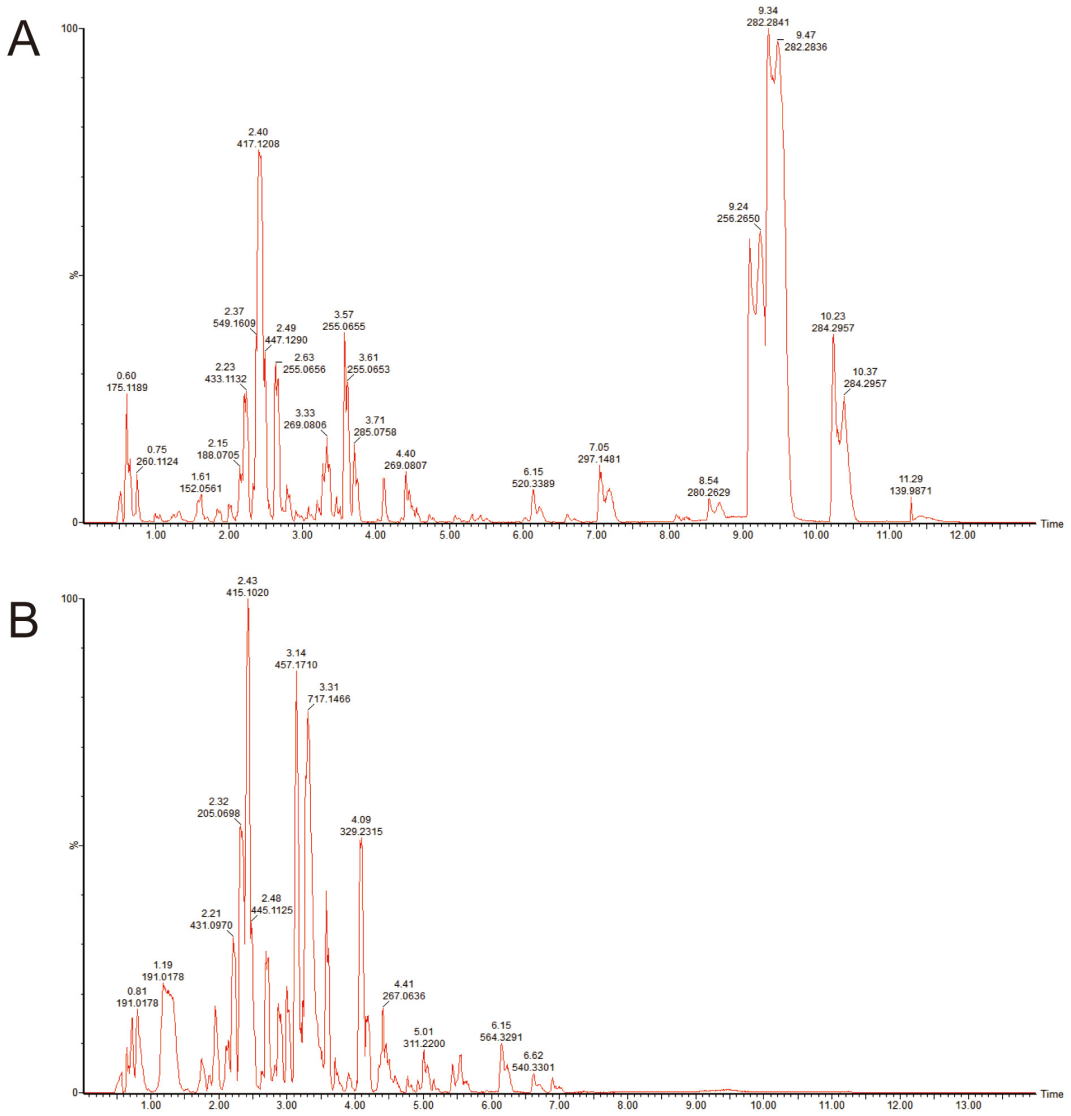
Ultra performance liquid chromatography - mass spectrometry of YQYYHX granules. **(A)** Mass spectrogram in positive ion mode. **(B)** Mass spectrogram in negative ion mode. The horizontal axis represents the time points of the reaction, while the vertical axis indicates the sum of the intensities of all ions.

YQYYHX granules from three different batches were analyzed by Ultra-performance Liquid Chromatography – Mass Spectrometry (Figure 2). Methanol (the extraction solvent) was used as the blank control. The raw data were processed using Progenesis QI 2.4 and subsequently analyzed using a self-built TCM database 2.0 developed by Beijing Junfei Technology Co., Ltd. The top 20 compounds with the largest peak area are shown in Table 2, such as daidzin, salvianolic acid L, and oleamide.

**Table 2:** The 20 compounds with the largest peak area in YQYYHX granules.

Number	Compound	m/z	Retention time (min)	Peak area of batch 1	Peak area of batch 2	Peak area of batch 3	Average peak area of three batches
1	Daidzin	417.12	2.42	53071727.22	52516740.65	55078672.30	53555713.39
2	Salvianolic acid L	717.15	3.30	24740672.84	24566021.34	25515061.76	24940585.31
3	Oleamide	282.28	9.36	31787758.35	16955158.52	14576010.89	21106309.25
4	Nordalbergin	255.07	3.57	18371379.08	18888206.30	19292409.60	18850664.99
5	Prunetin 4'-O-beta-D-glucopyranoside	447.13	2.49	15582195.49	15918850.44	16964987.64	16155344.53
6	Rubiadin primeveroside	549.16	2.37	13864726.60	13906458.78	13672893.43	13814692.94
7	Pulmatin	415.10	2.43	13784453.31	12941732.31	13303108.07	13343097.90
8	Aloe-emodin-8-o-beta-d-glucopyranoside	433.11	2.23	12791583.61	13325067.87	12601173.67	12905941.72
9	Puerarin	417.12	2.62	11951614.58	12073411.38	12506365.38	12177130.45
10	2-Hydroxy-4-allylphenyl 6-O-alpha-L-rhamnopyranosyl-beta-D-glucopyranoside	457.17	3.14	10772289.52	10844833.22	10061076.61	10559399.78
11	3-(1‘,1'-Dimethylallyl)Xanthyletin	297.15	7.06	10267816.25	10780094.15	10611122.83	10553011.08
12	1-Acetoxy-3-methoxy-9,10-anthraquinone	297.08	2.40	11062434.50	9049400.70	9552369.04	9888068.08
13	Lycopic acid C	521.11	3.27	8285120.42	8592400.24	9269637.94	8715719.53
14	L-Arginine	175.12	0.60	8472543.53	7727117.57	7966262.79	8055307.97
15	Isodalbergin	269.08	3.33	7312044.86	7278221.89	7420815.18	7337027.31
16	9S,12S,13S-Trihydroxy-10e-octadecenoic acid	329.23	4.10	7261367.80	7182788.16	6997099.83	7147085.27
17	7-Hydroxy-5,6-dimethoxy-1,4-phenanthrene-quinone	285.08	3.71	6476970.38	6472089.86	6512993.25	6487351.17
18	2-Methyl-N-phenylmaleimide	188.07	2.15	4735472.44	4575310.14	4489001.53	4599928.04
19	Daidzein	253.05	3.58	4469223.58	4534760.76	4669725.29	4557903.21
20	Vitexin	431.10	2.21	4726677.27	4489260.34	4438264.25	4551400.62

### Data management

All observations during the study must be recorded timely, accurately, completely, systematically, and truthfully. The original documents include outpatient medical records, medication administration records, subject diaries, and laboratory or examination reports. Confidential information will be accessible only to investigators and must not be disclosed in any publicly published materials.

Electronic case report forms are generated within the clinical record system, ensuring that all follow-up information is traceable. Baseline data, including sex, age, co-morbidities, concomitant medication, and other relevant characteristics, will also be recorded.

Then it will be entered into a specialized electronic data capture system for this trial. The data administrator will verify the entries and issue queries as needed. When queried, data entry personnel should review the original documents and provide appropriate responses. Then the data administrator will confirm and update the information to ensure consistency between the database and the original records.

### Outcomes

Participants who take the granules for more than 80% of the expected duration will undergo the following assessments before and after the 12-week treatment.

#### Primary outcome

Brachial–ankle pulse wave velocity (baPWV).

#### Secondary outcomes

Ankle–Brachial Index (ABI);

Fasting BG and HbA1c;

Lipid profiles;

Inflammatory cytokines;

6-min walk test;

Vascular Quality of Life Questionnaire-6 (VascuQol-6), symptom questionnaire, and TCM syndrome questionnaire (Tables S1–S3).

#### Safety outcomes

Electrocardiogram;

Complete blood count;

Liver and renal function tests;

Urinary albumin-to-creatinine ratio (UACR).

#### Exploratory mechanistic analysis

Serum and fecal samples from participants will be collected and stored at −80°C. After unblinding, serum samples from 30 participants in the treatment group will be used for metabolomic and proteomic sequencing, as well as other related analysis, while fecal samples will be used for metagenomic sequencing analysis. This study commits to handling all biological samples in strict accordance with regulatory requirements, ensuring that samples are not used for any unauthorized or unlawful research. All samples will be promptly destroyed after completion of the planned analyses.

Raw data will undergo quality control and appropriate preprocessing, followed by analysis using suitable statistical models, with multiple testing controlled by the Benjamini-Hochberg method. The identified molecular features will be further subjected to functional interpretation and exploratory integration with the clinical outcomes.

### Statistical plan

#### Analysis sets

The full analysis set (FAS) includes all participants who were randomized, using their last observed data as the trial outcome for intention-to-treat analysis. The per-protocol set (PPS) refers to participants who strictly adhered to the trial protocol, demonstrated good compliance, did not take prohibited medications during the trial, and completed the required case report forms. It will be utilized for the primary efficacy analysis. The safety set (SS) comprises all participants who were randomized, received at least one dose of the study treatment, and have post-treatment safety data available. Statistical descriptions and analyses of their safety outcomes and adverse event incidence will be performed.

#### Interim analysis

A group sequential design will be adopted for statistical inference, with a prespecified interim analysis to be conducted when approximately 50% of the total information for the primary endpoint has accrued. Interim analysis results and unblinded data will be accessible only to the independent Data Monitoring Committee, which will mainly consist of statisticians, clinical and ethics experts.

An O'Brien-Fleming efficacy boundary will be applied to control the overall two-sided type I error rate at 0.05, using the Lan-DeMets alpha-spending function as an approximation. Statistical significance will be determined on the basis of the prespecified nominal significance level corresponding to each analysis. If the baPWV crosses the prespecified efficacy boundary at an interim analysis, early termination of the trial may be recommended.

#### Outcome analysis

All data will be analyzed using SPSS 26.0 after unblinding. Continuous outcomes will be initially assessed for distributional characteristics by Shapiro-Wilk test. For approximately normally distributed outcomes, *t*-tests or ANCOVA will be applied to compare groups; for variables with marked deviation from normality, nonparametric alternatives such as the Mann–Whitney *U*-test and Wilcoxon signed-rank test will be considered. The categorical variables will be analyzed using the χ^2^ test.

Baseline covariates and other prognostic factors will be incorporated into ANCOVA models to improve estimation precision and control for residual confounding. Repeated measurements or longitudinal data will be analyzed using linear mixed-effects models, allowing for appropriate modeling of within-subject correlations, and evaluation of treatment-by-time interactions. The hierarchical testing procedure will be used to adjust multiple secondary outcomes. Sensitivity analysis will be conducted to reduce the impact of protocol deviations.

#### Multiple imputation

If any outcomes are randomly missing, multiple imputation will be used to enhance statistical power. Assuming that the missing data follow the Missing at Random assumption, the analysis results from multiple imputed datasets will be combined using Rubin's Rules. Methods such as comparison of distribution similarity, regression results, and Monte Carlo error assessment will be used to ensure the quality of imputation.

### Conditions for suspension, drop-out and termination

In accordance with medical standards, participant safety considerations, and GCP requirements, investigators will determine whether a participant needs to suspend or drop-out. Participants will be classified as drop-out if they are newly diagnosed with other diseases that may influence the trial, if they begin using other traditional Chinese medicines with similar therapeutic effects, or if they are lost to follow-up during the observation period.

An adverse event refers to any unfavorable medical occurrence during the study, regardless of whether it is causally related to the intervention. It includes clinically significant abnormalities in electrocardiogram, liver function, or renal function tests, as well as any subjective symptoms of discomfort reported by the participants. If it occurs, investigators should record, assess the severity and the relationship with the trial, and observe until returning to normal. A serious adverse event is any unfavorable medical occurrence that result in hospitalization, disability, incapacity, or even life-threatening conditions. It requires immediate medical management plus immediate reporting to the principal investigator once known, followed by expedited reporting to the ethics committee. If more than half of the participants experience serious adverse events, the trial must be terminated.

### Quality control

This trial is conducted in accordance with standard operating procedures for clinical trial management. All investigators have been authorized by the principal investigator, and have undergone appropriate training. A Trial Steering Committee is established to provide overall supervision of this study, and includes the principal investigator, as well as independent clinical and methodological experts. All granules are stored, managed, dispensed, and retrieved by professional pharmacists.

## Discussion

Our team has studied the formula and its main components for several years. We have found that Yiqihuoxue decoction (three herbs contained in YQYYHX) improved the BG, lipids, interleukin-6 (IL-6), tumor necrosis factor-α and aortic plaques in ApoE^−/−^ mice, while it increased the methylation of *AKT1, Nr1h3*, and *Fabp4* (30). Xiongshao Capsule (two herbs contained in YQYYHX) reduced the myeloperoxidase, total and free cholesterol in aorta wall cells of rabbits (31). Then we analyzed previous clinical prescriptions of the principal investigator and identified several herbs commonly used to treat patients with T2DM and EVA. Based on TCM theory and the above research foundation, we developed YQYYHX formula and prepared it as granules to improve the stability of its components. In unpublished studies, we also found that YQYYHX alleviated aortic plaques, reduced triglyceride and tumor necrosis factor-α in ApoE^−/−^ mice. Equol, a gut microbial metabolite derived from daidzein (one of the constituents of YQYYHX), has been reported to be associated with baPWV (32).

Arterial stiffness represents the risk of macrovascular complications in T2DM (33). PWV is used to evaluate arterial stiffness and vascular aging (34), defined as the ratio of the distance to the time traveled by the pulse wave between two specific sites. Although carotid-femoral PWV is commonly used for cardiovascular diseases, baPWV is easier and more convenient to test (35). It is used to measure the arterial stiffness of lower extremity arteries, whose normal range is below 1,400 cm/s. Age adjustment should be applied when assessing EVA. BaPWV is positively correlated with the risk of newly diagnosed diabetes in hypertensive patients (36). It is also positively correlated with aortic diameters of diabetic patients (37), while predicting risks of cardiovascular diseases (38). Patients with T2DM are more susceptible to coronary artery stenosis when baPWV exceeds 1,650 cm/s (39) and more susceptible to peripheral neuropathy when it exceeds 1,600 cm/s (40). It is positively correlated with heart rate, systolic blood pressure, triglyceride levels, and related indicators (41). Pathological baPWV is also correlated with 6MW distances (42) and UACR (43).

The normal range of ABI is 0.9–1.3. ABI < 0.9 indicates lower-extremity arterial stenosis or occlusion, while ABI > 1.3 indicates vascular calcification. ABI will decrease along with senescence (44), resulting in higher risks of cardiovascular events (45) and LEAD (46). As one of the key indicators for assessing glycemic control, HbA1c reflects average glycemic levels over the preceding 3 months. When it >7%, each 1% increase means 38% higher risk of macrovascular events of T2DM (47). It can also predict arterial stiffness (48). Lipid accumulation products have been shown to be positively correlated with baPWV (49). The triglyceride-glucose index also demonstrates a positive linear association with arterial stiffness (50, 51). As a characteristic of aging, systemic chronic inflammation can impair the clearance of senescent cells, weaken intrinsic tissue repair, promote immune-senescence, and induce cell death (52). Inflammation contributes to vascular aging by damaging vascular structures, disrupting functional homeostasis, and accelerating atherosclerosis. It is a marker of T2DM, EVA and atherothrombosis (53). IL-6 is a key cytokine implicated in promoting vascular cell senescence and senescence-associated mitochondrial dysfunction (54).

The 6 MW test serves as a valuable instrument for evaluating cardiopulmonary function. Specifically, it enables the assessment of ambulatory capacity, exercise tolerance, and muscle coordination in the lower extremities, with an opportunity to assess gait patterns. The 6 MW distance is negatively correlated with HbA1c levels, body mass index, and ages (55). VascuQol-6 is a simple and reliable instrument to assess the life quality of patients with intermittent claudication (56, 57). In our study, we also pay attention to changes in lower limb symptoms and TCM symptoms. Accordingly, we developed a symptom questionnaire and a TCM syndrome questionnaire by referencing relevant standards and guidelines. Patients with T2DM and EVA are more susceptible to renal damage (58). UACR is a relatively sensitive indicator for detecting early kidney injury.

This study provides an alternative and complementary therapy for patients with T2DM and EVA, who may benefit from it. This trial is designed with rigorous methodology and is expected to yield reliable results, providing high-quality evidence-based support (59) for clinical practice.

### Trial status

Until March 10, 2026, 72 participants have been enrolled, and 38 have completed the observation period.
