# Supplementary material for: Clinical effects of Yiqi-Yangyin-Huoxue granules in the management of type 2 diabetes mellitus and early vascular aging: a randomized, double-blind, placebo-controlled trial protocol
Source: Front Med (Lausanne). 2026 Jun 3;13:1768610. doi: 10.3389/fmed.2026.1768610 (PMC13273645; doi:10.3389/fmed.2026.1768610)
Supplement: Supplementary file 1 [file Table_1.docx]

Table S1. Vascular Quality of Life Questionnaire-6 (VascuQol-6)

|  | Question | criteria | score |
| --- | --- | --- | --- |
| 1 | Because of the poor circulation in my legs, the range of activities that I would have liked to do in the past two weeks has been… | 1. Severely limited - most activities not done  2. Very limited  3. Very slightly limited  4. Not limited at all - have done all the activities that I wanted to | 1□  2□  3□  4□ |
| 2 | During the past two weeks, my legs felt tired or weak… | 1. All of the time  2. Some of the time  3. A little of the time  4. None of the time | 1□  2□  3□  4□ |
| 3 | During the past two weeks, because of the poor circu lation in my legs, my ability to walk has been… | 1. Totally limited, couldn’t walk at all  2. Very limited  3. A little limited  4. Not at all limited | 1□  2□  3□  4□ |
| 4 | During the past two weeks, I have been concerned about having poor circulation in my legs… | 1. All of the time  2. Some of the time  3. A little of the time  4. None of the time | 1□  2□  3□  4□ |
| 5 | During the past two weeks, because of the poor circulation in my legs, my ability to participate in social activities has been… | 1. Totally limited, couldn’t socialize at all  2. Very limited  3. A little limited  4. Not at all limited | 1□  2□  3□  4□ |
| 6 | During the past two weeks, when I have had pain in the leg (or foot) it has given me… | 1. A great deal of discomfort or distress  2. A moderate amount of discomfort or distress  3. Very little discomfort or distress  4. No discomfort or distress | 1□  2□  3□  4□ |
| Total: | | | |
