# Supplementary material for: Clinical effects of Yiqi-Yangyin-Huoxue granules in the management of type 2 diabetes mellitus and early vascular aging: a randomized, double-blind, placebo-controlled trial protocol
Source: Front Med (Lausanne). 2026 Jun 3;13:1768610. doi: 10.3389/fmed.2026.1768610 (PMC13273645; doi:10.3389/fmed.2026.1768610)
Supplement: Supplementary file 2 [file Table_2.docx]

Table S2. Symptom questionnaire

| Symptoms | scores | criteria |
| --- | --- | --- |
| Limb feels cold | 4□  3□  2□  1□  0□ | 4: feels cold despite wearing more clothing  3: persistent coldness or feels cold despite wearing more clothing than normal  2: persistent coldness and relieved by wearing more clothing than normal  1: occasional  0: none |
| Limb soreness or distension | 4□  3□  2□  1□  0□ | 4: intolerable  3: persistent  2: occasional  1: soreness or distension when exercising more than normal  0: none |
| Limb numbness or stiffness | 4□  3□  2□  1□  0□ | 4: persistent stiffness  3: persistent numbness  2: occasional numbness or stiffness  1: numbness or stiffness when exercising more than normal  0: none |
| Intermittent claudication | 5□  4□  3□  2□  1□  0□ | 5: occurs immediately upon activity  4: occurs within 100 meters of walking  3: occurs more than 100 meters of walking  2: occurs more than 500 meters of walking  1: partly occurs more than 1000 meters of walking  0: no discomfort after walking more than 1500 meters |
| Rest pain | 4□  3□  2□  1□  0□ | 4: intolerable at rest, preventing sleep  3: tolerable pain  2: occasional pain  1: occurs after exertion  0: none |
| Burning sensation | 4□  3□  2□  1□  0□ | 4: intolerable and persistent  3: tolerable and persistent  2: occurring during the day or night  1: occasional  0: none |
| Total: | | |
