# Supplementary material for: Clinical effects of Yiqi-Yangyin-Huoxue granules in the management of type 2 diabetes mellitus and early vascular aging: a randomized, double-blind, placebo-controlled trial protocol
Source: Front Med (Lausanne). 2026 Jun 3;13:1768610. doi: 10.3389/fmed.2026.1768610 (PMC13273645; doi:10.3389/fmed.2026.1768610)
Supplement: Supplementary file 3 [file Table_3.docx]

Table S3. Traditional Chinese medicine (TCM) syndrome questionnaire

| Symptoms | | scores | criteria |
| --- | --- | --- | --- |
| Primary symptoms | thirsty | 6□  4□  2□  0□ | 6:  intolerable, red throat, frequent drinking  4:  tolerable, slightly red throat, occasionally desire to drink  2: a little thirsty and mild throat dryness, no need to drink  0: none |
|  | Shortness of breath with reluctance to speak | 6□  4□  2□  0□ | 6: unwilling to speak  4: reluctant to speak  2: speak little  0: none |
|  | Limb numbness or stiffness | 6□  4□  2□  0□ | 6: severe  4: moderate  2: mild  0: none |
|  | Stabbing pain in the distal limbs | 6□  4□  2□  0□ | 6: severe pain, severely affects daily activities  4: moderate pain, moderately affects daily activities  2: mild pain, not affects daily activities  0: none |
| Secondary symptoms | Limb weakness | 3□  2□  1□  0□ | 3: severe weakness, not relieves by rest, limits daily activities  2: moderate weakness, relieves by rest  1: mild weakness, not affect daily activities  0: none |
|  | Spontaneous sweating and night sweats | 3□  2□  1□  0□ | 3: sweating without activity, and sweating very more after activities  2: slightly damp skin at rest, worsened with minor movement  1: mild sweating after activity  0: none |
|  | Heat sensation in the palms and soles | 3□  2□  1□  0□ | 3: relieved by holding cold objects  2: desire to expose hands and feet without clothes  1: mild sensation  0: none |
|  | Palpitations and insomnia | 3□  2□  1□  0□ | 3: frequently  2: occasionally  1: rarely  0: none |
|  | Dried skin | 3□  2□  1□  0□ | 3: Skin is thickened and rough with pigmentation, looks like dried fish scales  2: cracks or grooves on the skin surface  1:  dry and rough skin  0: none |
|  | Cyanotic lips | 3□  2□  1□  0□ | 3: severe  2: moderate  1: mild  0: none |
|  | Constipation or diarrhea | 3□  2□  1□  0□ | 3: frequently  2: occasionally  1: rarely  0: none |
| Total: | | | |
